# Playing with covalent triazine framework tiles for improved CO_2_ adsorption properties and catalytic performance

**DOI:** 10.3762/bjnano.10.121

**Published:** 2019-06-12

**Authors:** Giulia Tuci, Andree Iemhoff, Housseinou Ba, Lapo Luconi, Andrea Rossin, Vasiliki Papaefthimiou, Regina Palkovits, Jens Artz, Cuong Pham-Huu, Giuliano Giambastiani

**Affiliations:** 1 Institute of Chemistry of OrganoMetallic Compounds, ICCOM-CNR and Consorzio INSTM, Via Madonna del Piano 10-50019, Sesto F.no, Florence, Italy; 2 Institut für Technische und Makromolekulare Chemie RWTH Aachen University, Worringerweg 2, D-52074, Aachen, Germany; 3 Institut de Chimie et Procédés pour l’Energie l’Environnement et la Santé (ICPEES) UMR 7515 CNRS University of Strasbourg (UdS) 25 rue Becquerel 67087, Strasbourg Cedex 02, France; 4 Kazan Federal University, Kremlyovskaya Str. 18, 420008 Kazan, Russia

**Keywords:** covalent triazine frameworks, CO_2_ adsorption, CO_2_/N_2_ selectivity, dehydrogenation catalysis, ionothermal conditions

## Abstract

The rational design and synthesis of covalent triazine frameworks (CTFs) from defined dicyano-aryl building blocks or their binary mixtures is of fundamental importance for a judicious tuning of the chemico-physical and morphological properties of this class of porous organic polymers. In fact, their gas adsorption capacity and their performance in a variety of catalytic transformations can be modulated through an appropriate selection of the building blocks. In this contribution, a set of five CTFs (**CTF1**–**5**) have been prepared under classical ionothermal conditions from single dicyano-aryl or heteroaryl systems. The as-prepared samples are highly micro-mesoporous and thermally stable materials featuring high specific surface area (up to 1860 m^2^·g^−1^) and N content (up to 29.1 wt %). All these features make them highly attractive samples for carbon capture and sequestration (CCS) applications. Indeed, selected polymers from this series rank among the CTFs with the highest CO_2_ uptake at ambient pressure reported so far in the literature (up to 5.23 and 3.83 mmol·g^−1^ at 273 and 298 K, respectively). Moreover, following our recent achievements in the field of steam- and oxygen-free dehydrogenation catalysis using CTFs as metal-free catalysts, the new samples with highest N contents have been scrutinized in the process to provide additional insights to their complex structure–activity relationship.

## Introduction

Recent years have witnessed an increasing interest in carbon-based nanomaterials as functional devices for energy-related applications [1]. Their unique properties, such as their semiconducting behaviour, their inherent porosity, high specific surface area, chemical versatility, including their thermal and chemical resistance make them ideal candidates for a number of energy storage and conversion technologies [2–3]. The scope of carbon-based nanomaterials therefore covers a wide range of applications in (photo-/electro-)catalysis, gas storage and separation technologies as well as energy storage devices. Among nanocarbons, (nano)porous organic polymers (POPs) have gained a significant popularity because of their unique features [4–8]. Indeed, the use of a wide variety of rigid and sterically demanding organic building blocks to synthesize POPs allows for a fine control of their morphological and chemical properties [9–11]. Thus, POPs provide a permanent porosity (with high accessible specific surface area), combined with a facile chemical modification, e.g., the inclusion of heterocycles and light elements within the organic functional units.

Covalent triazine frameworks (CTFs) represent a POP subclass of highly crosslinked porous polymers, generated by the cyclotrimerization of dicyano-(hetero)aryl building blocks [12–13]. Under ionothermal conditions, in molten zinc chloride, the rational combination of dicyano-substituted organic moieties can be used to provide stable carbon nanomaterials with diverse morphologies (i.e., porosity and specific surface area) along with variable chemical composition (i.e., content and type of light elements such as N, S and O) [14–15]. Major application fields of CTFs are represented by energy storage and conversion [16–18], gas storage and separation (e.g., H_2_, CO_2_ and CH_4_) [19–21] as well as various catalytic uses [22–30].

The exceptional performance of CTFs in capture and storage of CO_2_ has prompted us to further exploit their potentiality in that direction through a judicious tuning of their ultimate structural and chemical properties. While the gas-storage capacity of a solid is mainly influenced by its porosity and accessible surface area [31–32], the Lewis basicity of its surface generates preferential interactions with Lewis acids such as CO_2_ [33]. The rational selection of monomers featured by Lewis-basic sites, eventually combined with structural directing co-monomers can be used to tune the surface basicity and morphology of the materials and, consequently, optimize their gas-adsorption capacity. In addition, the control of the chemico-physical properties (i.e., pore-size distribution, specific surface area (SSA) and surface basicity) of the target samples is known to play a fundamental role in the control of their performance (activity and stability) as metal-free catalysts in gas-phase processes. Our recent achievements in the use of highly porous and N-rich carbon nanomaterials as metal-free catalysts for the steam- and oxygen-free dehydrogenation catalysis (DDH) of ethylbenzene (EB) to styrene (ST) have shown unique outcomes in terms of specific process rate (λ) and ST selectivity, even under operative conditions close to those of industrial plants [34]. Among these, CTFs have unambiguously exhibited superior activity and selectivity in the process [30] compared to carbon-based and metal-based state-of-the-art systems [35–44]. Most importantly, the rational balance between morphological and basic material surface properties has been claimed to control the catalyst stability on stream: the higher the “chemically accessible” surface basicity, the lower the sample deactivation/passivation due to the generation of coke deposits [30].

**Scheme 1:**
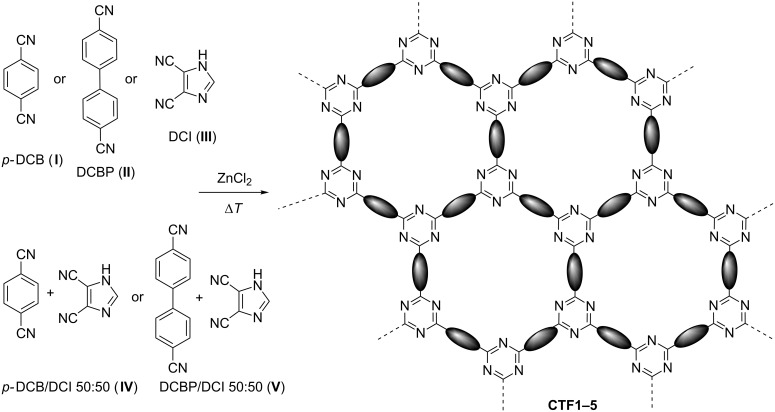
Idealized ionothermal synthesis of **CTF1**–**5** from 1,4-dicyanobenzene (*p*-DCB, **I**), 4,4′-dicyanobiphenyl (DCBP, **II**), 4,5-dicyanoimidazole (DCI, **III**) as well as from their equimolar mixtures (*p*-DCB/DCI, **IV**, and DCBP/DCI, **V**).

This contribution describes the synthesis and characterization of two model CTFs based on 1,4-dicyanobenzene (*p*-DCB) and 4,4′-dicyanobiphenyl (DCBP) and their comparative analysis in terms of chemico-physical properties with newly synthesized samples derived from 4,5-dicyanoimidazole (DCI) or its equimolar mixtures with the aforementioned dicyanoaryl units (see Scheme 1 below) [45]. The as-prepared samples have been investigated as CO_2_ storage materials as well as metal-free catalysts for the gas-phase DDH of EB to ST. Notably, mixed CTF samples from this series have shown CO_2_ adsorption capacities that rank among the highest reported so far in the literature. Furthermore, an ideal combination of material morphology and chemical composition has provided a sample that largely outperforms the classical benchmark carbon materials in terms of DDH catalytic performance (activity and ST selectivity) as well as stability on stream.

## Results and Discussion

### Synthesis and characterization of **CTF1–5**

CTF samples have been prepared under ionothermal conditions, using molten ZnCl_2_ as reaction medium and Lewis acid cyanotrimerization catalyst [14]. As ZnCl_2_ is supposed to act as a porogene, it was used in large excess with respect to the monomer (ZnCl_2_/monomer = 5:1 molar ratio). After a sequential heating of the monomer/salt mixture at 400 °C and 600 °C for 10 + 10 h in sealed quartz ampules, CTFs have been isolated as amorphous and partially carbonized frameworks. The as-prepared samples feature high specific surface areas showing variable N loadings and N configurations as a function of the type of monomer(s) used. X-ray powder diffraction analyses have confirmed, as expected, the substantially amorphous nature [19,33] of all CTF samples from this series (Supporting Information File 1, Figure S1). Scheme 1 summarizes the different building blocks employed for the synthesis of CTFs in this work, while Table 1 lists all their main chemico-physical and morphological properties. Materials obtained from 1,4-dicyanobenzene (*p*-DCB, **I→CTF1**) and 4,4`-dicyanobiphenyl (DCBP, **II→CTF2**) show isotherm profiles typical of bimodal micro-mesoporous materials with complex and ill-defined pore networks (see Supporting Information File 1, Figures S2A,A′ and S2B,B′). As found for related CTF samples previously synthesized by us under similar reaction conditions [30], **CTF2** presents a type-IV isotherm profile with a distinctive H2 hysteresis loop in the range of *p*/*p*_0_ = 0.4–0.6. As expected from its longer linker, **CTF2** shows an increase of mesoporosity with respect to **CTF1** (mesopore volume from 60% to 75% of the total pore volume).

**Table 1:** Specific surface area, pore size distribution and N content (wt % loading and % of each N configuration) as measured for **CTF1**–**5**.

entry	sample	SSA^a^ [m^2^ g^−1^]	*V*_p(total)_^b^ [cm^3^ g^−1^]	*V*_p(micro)_^c^ [cm^3^ g^−1^]	*V*_p(micro)_^c^ [%]	*N*^d,e^ [wt %]	pyridinic N %^f^	pyrrolic N %^f^	oxidic N %^f^

1	**CTF1**	1654	1.06	0.42	40	7.5	39	51	10
2	**CTF2**	1863	1.31	0.33	25	3.6	42	50	8
3	**CTF3**	352	0.19	0.19	100	29.1	58	36	6
4	**CTF4**	784	0.41	0.30	73	18.1	51	42	7
5	**CTF5**	1489	0.80	0.44	55	11.4	43	46	11

^a^Brunauer–Emmett–Teller (BET) specific surface area (SSA) measured at 77 K. ^b^Total pore volume determined by using the adsorption branch of N_2_ isotherm at *p*/*p*_0_ = 0.98. ^c^Micropore volume calculated by a NLDFT model. ^d^Determined by elemental analysis as average values from three independent measurements. ^e^Complete CHN elemental analyses of the five CTF samples are given in Supporting Information File 1, Table S1. ^f^Determined by XPS analyses.

Both samples present a high and comparable specific surface area and a total pore volume up to 1.31 cm^3^·g^−1^ (Table 1, entries 1 and 2). Although their structural properties sound promising for gas-adsorption applications, their N content remains moderate. As N content and related surface basicity play a key role in the CO_2_ adsorption capacity of CTF samples, we have focussed on 4,5-dicyanoimidazole (DCI, **III**) as a novel and highly N-rich monomer to be used for CTF synthesis as such (**III**→**CTF3**), or in equimolar mixture with one of the two other building blocks (**IV**→**CTF4**; **V**→**CTF5**).

N_2_ physisorption isotherms recorded on **CTF3**–**5** present classical type-I(b) profiles [46] that basically account for samples with a prevalent microporous structure (see Table 1, entries 3–5 and Supporting Information File 1, Figure S2C–E and Figure S2C′–E′). In spite of its purely microporous nature and moderate SSA (352 m^2^·g^−1^), **CTF3** holds one of the highest N contents (29.1 wt %) reported so far in the literature for CTF prepared via ionothermal synthesis. Accordingly, the use of DCI monomer (**III**) in combination with **I** or **II** has been exploited to obtain materials that combine high specific surface area, high mesopore density and high N loading. For both mixed CTFs (**CTF4** and **CTF5**), monomer **III** has been used in equimolar amount with either **I** or **II**, while keeping the ZnCl_2_/monomer(s) molar ratio constant at 5:1. Isotherms recorded on mixed CTFs (**CTF4**,**5**) account for materials with markedly higher gas-uptake capacities compared to **CTF3**. Indeed, the use of a co-monomer for **III** in the cyanotrimerization step is found to double or quadruple the specific surface areas and total pore volumes on the corresponding CTFs (Table 1, entry 3 vs entries 4 and 5). Moreover, **CTF4**,**5** show a N content that is much higher than that of materials prepared from pure monomers **I** and **II**. As expected, the greater the size of the *para*-dicyano aryl co-monomer, the greater the share of mesopores (%) and their size distribution in the target material. Indeed, **CTF5** (DCBP/DCI) holds a percentage of mesopores up to 18% higher than its counterpart **CTF4** (*p*-DCB/DCI) and mesopore sizes up to 40 Å (Table 1 and Supporting Information File 1, Figures S2D–E and D′–E′).

The N 1s XPS spectra recorded for all new CTF samples are fitted with two main components and a minor shoulder at binding energies (BE) between 398.5 ± 0.2, and 402.5 ± 0.5 eV. (see Supporting Information File 1, Figure S3A–E and Figure S4). While the former component at lower BEs is unambiguously ascribed to pyridinic N atoms from both triazine frameworks and the pyridinic N sites of the imidazole groups, peaks centred at 400.5 ± 0.2 are likely due to pyrrolic N species mostly deriving from a partial decomposition/rearrangement of the samples during thermal treatment [30,47–49]. Minor shoulders at higher binding energies for all N 1s profiles are finally attributed to a certain extent of N–O species in the samples [50] (Table 1). Notably, all materials prepared from the DCI (**III**) monomer as such (**CTF3**) or in mixture with *p*-DCB (**I**) (**CTF4**) or DCBP (**II**) (**CTF5**) maintain a relatively high percentage of pyridinic nitrogen (Table 1 and Supporting Information File 1, Figure S3A–E). Such a result is reasonably ascribed to a higher thermal stability of the *ortho*-dicyano monomer **III** compared to the *para*-dicyano aryl systems **I** and **II** under the ionothermal conditions.

### CO_2_ adsorption properties of **CTF1–5**

**Figure 1:**
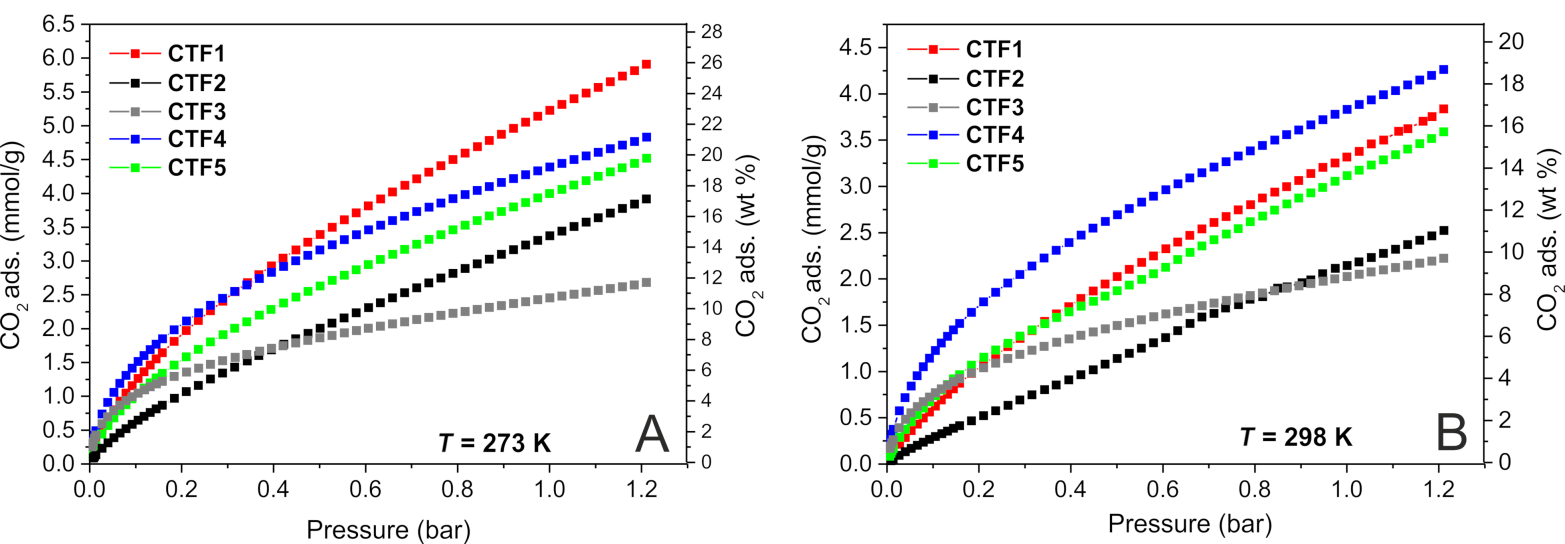
Low-pressure CO_2_ isotherms for **CTF1** (red), **CTF2** (black), **CTF3** (grey), **CTF4** (blue) and **CTF5** (green) measured at A) *T* = 273 K and B) *T* = 298 K. CO_2_ desorption curves are not reported in here for the sake of clarity. Complete adsorption–desorption isotherms are reported in Supporting Information File 1, Figure S5A,B.

The wide morphological and chemical diversity of the as-synthesized CTF samples prompted us to evaluate their CO_2_ adsorption and separation capacities. To this aim, all materials were firstly activated under ultrahigh vacuum and CO_2_ isotherms were recorded at *T* = 273 K and *T* = 298 K, in order to calculate the CO_2_ heat of adsorption (*Q*_st_). All these data are summarized in Table 2 and systematically compared with those reported in the literature for related CTF systems. As Figure 1 shows, neither purely microporous, although highly N-rich, samples (**CTF3**) nor mesoporous and N-poor solids (**CTF2**) were ideal candidates for the CO_2_ capture and storage.

Among the prepared CTFs, **CTF1** and **CTF4** exhibit a CO_2_ adsorption uptake at ambient pressure as high as 5.23 and 3.83 mmol·g^−1^ at 273 and 298 K, respectively. A comparative analysis with the current literature data (according to samples analyzed under similar pressure and temperature conditions, see Table 2) reveals that **CTF1** and **CTF4** rank among the samples with the highest CO_2_ uptake capacity reported up to now in the literature both at *T* = 273 and 298 K. With the exception of the CFT-py^HT^ sample [30] (featured by a markedly higher specific surface area of 3040 m^2^·g^−1^; Table 2, entry 9), the highly N/O co-doped HAT-CTF material [51] (1090 m^2^·g^−1^; Table 2, entry 15) and the perfluorinated *df*-TzCTF600 [52] (1720 m^2^·g^−1^; Table 2, entry 28), **CTF4** outperforms the CO_2_ adsorption capacity of many benchmark systems from this class of porous organic polymers. With 1.23 mmol·g^−1^ and 3.83 mmol·g^−1^ of adsorbed CO_2_ at room temperature and 0.1 bar and 1 bar pressure, respectively (Table 2, entry 4), **CTF4** surpasses the adsorption ability of samples such as bipy-CTFs (3.07–2.95 mmol·g^−1^; Table 2, entries 10, 11) [33], F-CTF (3.21–3.41 mmol·g^−1^; Table 2, entries 13, 14) [53], PHCTFs (1.57–1.34 mmol·g^−1^; Table 2, entries 17, 18) [54], bpim-CTFs (2.46–2.77 mmol·g^−1^; Table 2, entries 22, 23) [55] CTF-CSU41 (1.80 mmol·g^−1^; Table 2, entry 24) [56], PHCTF-8(650) (2.54 mmol·g^−1^; Table 2, entry 25) [57] and acac-CTF-5-500 (1.97 mmol·g^−1^; Table 2, entry 27) [58].

**Table 2:** CO_2_ adsorption uptake, isosteric heat of adsorption (*Q*_st_) and CO_2_/N_2_ selectivity values measured for **CTF1**–**5** at comparison with the most representative CTF systems from the literature.

entry	sample	CO_2_ uptake (mmol·g^−1^)				*Q*_st_ (kJ·mol^−1^)	CO_2_/N_2_ selectivity		ref.
		*T* = 273 K		*T* = 298 K					
		0.1 bar	1 bar	0.1 bar	1 bar		Henry	IAST	

1	**CTF1**	1.27	**5.23**	0.62	3.32	34.0	13	11	this work
2	**CTF2**	0.65	3.37	0.30	2.14	32.8	10	9	this work
3	**CTF3**	1.05	2.46	0.77	2.03	25.8	59	65	this work
4	**CTF4**	1.51	4.39	**1.23**	**3.83**	21.5	75	46	this work
5	**CTF5**	1.05	4.00	0.74	3.12	24.9	25	19	this work
6	CTF-ph	1.13	4.54	0.58	3.05	33.2	20	—	[30]
7	CTF-ph^HT^	0.66	4.17	0.36	2.69	25.4	11	—	[30]
8	CTF-py	2.03	5.08	1.12	3.79	35.1	45	—	[30]
9	CTF-py^HT^	1.04	5.97	0.61	4.22	27.1	29	—	[30]
10	*bipy*-CTF500	—	5.34	—	3.07	34.2	61	42	[33]
11	*bipy*-CTF600	—	5.58	—	2.95	34.4	37	24	[33]
12	*fl*-CTF350	—	4.28	—	2.29	32.7	27	23	[19]
13	F-CTF-1	1.76	4.67	0.92	3.21	35.0	—	31	[53]
14	F-CTF-1-600	1.40	5.53	0.68	3.41	32.0^b^	—	19	[53]
15	HAT-CTF-450/600	3.0^a^	6.3	2.0^a^	4.8	27.1	126	110	[51]
16	caCTF-1-700	—	6.00	—	3.55	30.6	—	—	[59]
17	PHCTF-4	—	2.34	—	1.57	34.5^b^	40	35	[54]
18	PHCTF-5	—	2.18	—	1.34	32.5^b^	67	138	[54]
19	CTF-20-400	—	3.48	—	2.09	22	19	—	[60]
20	CTF-5-500	—	3.02	—	1.90	26	36	—	[60]
21	F-DCBP-CTF-1	2.15^a^	5.98	1.19^a^	3.82	33.1	—	31	[61]
22	bpim-CTF400	—	–	—	2.46	31	—	32	[55]
23	bpim-CTF500	—	–	—	2.77	28	—	24	[55]
24	CTF-CSU41	—	2.34	—	1.80^b^	44.6	—	35.3	[56]
25	PHCTF-8(650)	1.30^a^	4.00	—	2.54	28	56	89	[57]
26	CTF-BIB-1	—	4.35	—	—	35.2	—	29.3	[62]
27	acac-CTF-5-500	—	3.30	—	1.97	28.6	46	—	[58]
28	*df*-TzCTF600	2.17^a^	6.79	—	4.60	34	21	30	[52]
29	CTF-TPC	—	4.24	—	2.69	32	20^c^	30^c^	[20]
30	MM2	—	4.70	—	3.13	32	23^c^	44^c^	[21]

^a^Measured at 0.15 bar. ^b^Estimated value from the low-pressure CO_2_ isotherms in the original paper. ^c^Calculated at 273 K.

**CTF1** presents the highest CO_2_ adsorption capacity at 1 bar pressure among the samples of this series when analyses are carried out at the lower temperature (273 K). Under these conditions, the adsorption gap with related samples from the literature (Table 2, entry 1 vs entries 6–28) appears somewhat reduced. Anyhow, the relatively high SSA and N content of **CTF1** together with its micro-mesoporous morphology (see Table 1, entry 1) keep it among the samples with the highest CO_2_ uptake values claimed so far for this class of materials.

To better specify the binding affinity between **CTF1**–**5** and CO_2_, the isosteric heat of adsorption (*Q*_st_) has been calculated from the CO_2_ isotherms recorded for each material at *T* = 298 and 273 K, using a variant of the Clausius–Clapeyron equation [63] (see Supporting Information File 1, Figure S6). Such a measurement strongly relies on the morphological and chemical properties of the material and it is generally claimed to reflect the interaction strength between CO_2_ and the sorbent samples [30]. However, the *Q*_st_ values and the CO_2_ adsorption capacity on porous samples do not always coherently correlate [64]. Indeed, the literature presents several examples of materials featuring very high *Q*_st_ values but only moderate CO_2_ uptake [54,56]. The *Q*_st_ value of **CTF3** is relatively high because of its exceptionally high N content. However, its adsorption capacity is markedly reduced compared to **CTF1** and **CTF2** (Table 1, entry 3 vs entries 1 and 2) because of its markedly decreased pore volume (the total pore volume of **CTF3** is roughly reduced to one tenth compared to **CTF1** and **CTF2**). At odds with its high adsorption capacity (3.83 mmol·g^−1^, *T* = 298 K, 1 bar of CO_2_), the *Q*_st_ value of **CTF4** (21.5 kJ·mol^−1^) is lower compared to its congeners. Similarly, the pore volume of **CTF5** is higher than that of **CTF4** while the N loading is smaller; this translates in comparable *Q*_st_ values for the two mixed samples (Table 2, entries 4 and 5). Overall, *Q*_st_ values measured for CTF samples from this series fall in the ideal range for CO_2_ storage materials (<40 kJ·mol^−1^) [52], which ensures lower regeneration cost compared to widely used amine solutions (>40 kJ·mol^−1^) [65] for CCS targets.

Given the CO_2_ affinity of the materials, and in view of their practical application as CO_2_ adsorbents from gas mixtures, the competitive CO_2_ uptake over N_2_ has been determined for each sample using the ratio of the initial slopes in the Henry region of the adsorption isotherms (CO_2_ and N_2_) at *T* = 298 K (see Supporting Information File 1, Figure S7). The resulting values (listed in Table 2) generally come from the combination of two distinct material features: (i) adsorption selectivity and (ii) uptake capacity [52]. Accordingly, the values range from moderate (**CTF1**,**2** and **CTF5**) to relatively high in the case of **CTF3** and **CTF4**. For the sake of completeness, the selective CO_2_ capture from CO_2_/N_2_ mixtures was additionally calculated using the simplified ideal adsorbed solution theory (IAST) model [66]. Accordingly, ideal selectivity values of CO_2_ over N_2_ were calculated (Table 2) at an equilibrium partial pressure of 85% N_2_ and 15% CO_2_ in the bulk phase by combining the experimental single-component isotherms. Among the CTFs of this study, the DCI derivative (**CTF3**) gives an ideal selectivity value of 65 that is the highest calculated for this material series. This result is in line with the pure microporous nature of the sample whose channels match better with the CO_2_ dimensions providing a higher kinetic selectivity for CO_2_ separation from CO_2_/N_2_ mixtures [56].

### **CTF1–5** as metal-free catalysts

From the viewpoint of sustainable technologies, the heterogeneous catalysis with complex carbon networks as metal-free systems, including carbon matrices hetero-doped with light elements, has received a great deal of interest from several research groups operating in the area of industrially relevant transformations. CTFs have been recently reported by some of us as highly stable and effective heterogeneous systems for promoting a challenging transformation such as the steam- and oxygen-free dehydrogenation (DDH) of ethylbenzene (EB) to styrene (ST) [30]. We demonstrated how the unique DDH performance of selected CTFs was the result of a compromise between morphological and accessible basic surface properties of the samples. In particular, the higher the chemically accessible basic surface of the catalytic materials, the higher their stability (as catalysts) on stream. Indeed, from the comparative analysis of CTFs featuring different chemical and morphological properties, we postulated the existence of a close relationship between the rate of cracking side reactions leading to catalyst deactivation (formation of coke deposits) and the kinetic desorption of reagents and products from the catalyst surface. The higher the basic surface properties of the CTF, the higher the desorption rate of reagents and products and the higher the catalyst lifetime on stream. This evidence is in line with other findings from the literature where basic properties of the material are crucial in preventing the occurrence of side cracking reactions of EB [34–35,37–38,67–70].

**Figure 2:**
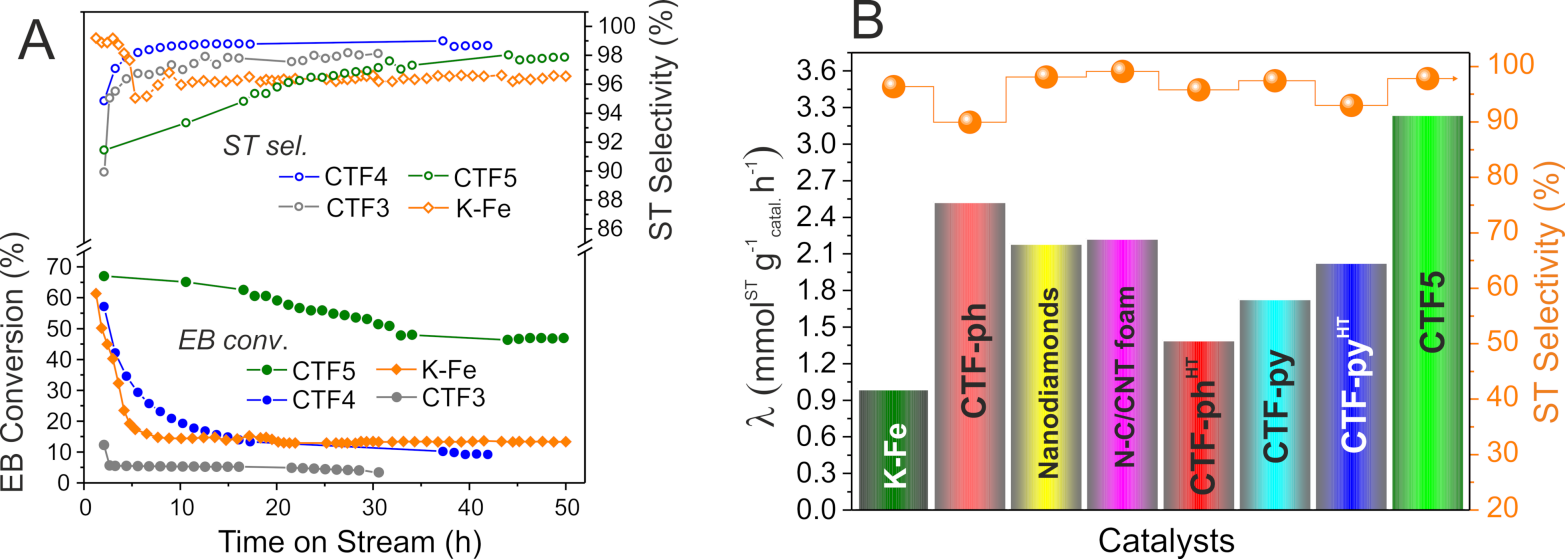
A) DDH of EB with **CTF3** (filled grey spheres), **CTF4** (filled blue spheres), and **CTF5** (filled green spheres) along with the respective ST selectivity: ST sel. of **CTF3** (open grey spheres); ST sel. of **CTF4** (open blue spheres), and ST sel. of **CTF5** (open green spheres). DDH performance of the benchmark K–Fe catalyst, EB conv. (filled orange diamonds) and ST sel. (open orange diamonds) are reported under identical reaction conditions for the sake of comparison. B) Catalytic performance (specific reaction rate λ (coloured histograms) and ST selectivity (orange spheres)) for **CTF5** in comparison with the most representative CTF samples from the literature [30] along with state-of-the-art carbon-based catalysts (N–C/CNT foam and NDs taken from [34]). The K–Fe_2_O_3_ catalyst is reported for the sake of comparison. All values of λ and ST selectivity are measured under steady-state conditions, after 30 h on stream except for **CTF5** the λ value of which was measured after 50 h on stream. Experimental conditions: catalyst 300 mg; *T* = 550 °C; 2.8% EB in He at 30 mL·min^−1^.

With a view to the N loading and N configuration of the newly synthesized samples, **CTF3**–**5** offer a variety of morphological properties (from purely microporous to micro-mesoporous samples) along with high charges of nitrogen (up to 29.1 wt %) and, in particular, basic N sites (N_Py_) (from 43 to 58%) (Table 1, entries 3–5). Hence, the three CTF samples have been scrutinized for the DDH of EB to ST and their performance, in terms of EB conversion (filled circles) and ST selectivity (empty circles), are outlined in Figure 2 and compared to the industrially used K–Fe catalyst under identical conditions (550 °C, 2.8 vol % EB diluted in He, total flow rate: 30 mL/min).

**CTF5** outperformed the other two metal-free systems as well as the benchmark K–Fe catalyst under steady-state conditions. Despite its remarkably high N content, **CTF3** shows a very moderate performance on stream with an EB conversion that roughly lies around 5% and a ST selectivity close to 98%. Such a result is likely ascribed to its purely microporous nature that largely prevents the regular EB uptake to the bulk active sites for the process to occur.

CTFs featuring larger mesopore domains (Table 1, entry 3 vs entries 4 and 5), albeit providing a lower number of N sites (**CTF4** and **CTF5**), show good to excellent catalyst performance. **CTF4** performs similarly to K–Fe in terms of EB conversion, showing a largely superimposable profile to that of the benchmark system under identical conditions. Despite a higher ST selectivity compared to its metallic counterpart (98.5 vs 96.4% after 40 h on stream), **CTF4** shows a rapid deactivation already within the first hours on stream that progressively continues (although more slowly) after 20 h on stream, thus revealing its rather moderate stability. Such a moderate EB conversion (at the steady-state) and rapid catalyst deactivation on stream is ascribed to pore clogging caused by the formation of coke deposits that progressively reduces the access of EB to the active sites. With an increased volume of the mesoporous component (Table 1, entry 5 vs entry 4) and a relatively high N content (up to 11.4 wt %), **CTF5** largely outperforms all CTFs from this series and exhibits a catalytic performance that is the highest reported so far for a metal-free catalyst in DDH under steam- and O_2_-free conditions. The absence of a marked deactivation of this sample in the first hours on stream is distinctive for an open-cell pore structure where the chemically accessible basic character of the material (due to the presence of a relatively high fraction of basic N sites) is supposed to reduce the occurrence of side processes responsible for the progressive catalyst passivation on stream. The catalyst stabilizes in the first 30 h on stream during which EB conversion gradually decreases (although it always remains over 50%) and then floats almost constantly around (43 ± 0.5)% for the remaining time (till 50 h). ST selectivity gradually increases to approximately 98% after 50 h on stream. Under these conditions, the measured specific reaction rate (λ), expressed as the amount of ST obtained per gram of catalyst per hour at the steady state is 3.24 (mmol^ST^·g^−1^_Catal_·h^−1^). Such a value certainly ranks among the highest rates claimed so far for CTFs as well as for various metal-free C-networks applied to the process (Figure 2B). The λ value measured for **CTF5** is even higher than that recorded for selected classes of mesoporous carbon nanomaterials, i.e., nanodiamonds (NDs) [39–40] and 3D foams (N–C/CNT) [34], that are commonly quoted as benchmark metal-free systems for DDH. Finally, temperature-programmed oxidation (TPO) analyses have been conducted on the fresh and spent CTF samples used in long-term catalytic DDH runs. As Supporting Information File 1, Figure S8 shows, the TPO profiles of **CTF5** before and after catalysis (see Figure 2A, after 50 h on stream and Figure S8B, Supporting Information File 1) are largely superimposable with a slight increase of the low-temperature component only. On the other hand, the TPO profile of the spent **CTF4** presents (see Figure 2A, after 40 h on stream and Figure S8A, Supporting Information File 1) an evident peak enlargement due to a non-negligible formation of low-temperature carbon deposits (coke). These results mirror the different catalytic behaviour of the two CTF systems at work in DDH and highlight the higher stability of the highly basic and open-cell-structured **CTF5** sample under operative conditions.

## Conclusion

The rational combination of highly N-rich building blocks for the bottom-up synthesis of highly porous organic polymers with improved CO_2_ adsorption properties has prompted us to explore the generation of mixed covalent triazine frameworks. The ionothermal synthesis of mixed CTFs from equimolar mixtures of 4,5-dicyanoimidazole (DCI, **III**) and 1,4-dicyanobenzene (*p*-DCB, **I**) or 4,4′-dicyanobiphenyl (DCBP, **II**), has provided amorphous polymers with variable (from moderate to high) specific surface areas and bimodal micro-mesoporous morphologies. In particular, the greater the size of the *para*-dicyano aryl co-monomer (**I** or **II**), the greater the mesopore component (%) in the target mixed material. The use of a co-monomer for **III** in the cyanotrimerization step doubles or quadruples the specific surface area and total pore volume of the resulting mixed-CTF samples compared to the material prepared from the unique monomer **III**. In addition, mixed CTFs exhibit a higher N loading than the samples obtained from the pure monomers **I** and **II**. With 1.23 mmol·g^−1^ and 3.83 mmol·g^−1^ of adsorbed CO_2_ at room temperature and 0.1 bar and 1 bar pressure, respectively, **CTF4** ranks among the benchmark systems for this class of materials. In addition, the mesoporous nature of the N-rich mixed sample **CTF5** has been found to fulfil ideally the key morphological and chemical requirements for a highly robust and active catalyst for the dehydrogenation of ethylbenzene to styrene. **CTF5** has shown excellent performance as a metal-free catalyst in the process, working under steam-and O_2_-free conditions. With a specific reaction rate, λ, of 3.24 (mmol^ST^·g^−1^_Catal_·h^−1^) under steady-state conditions and with a markedly high stability on stream, **CTF5** outperforms materials from the same sample series as well as various doped and undoped C-networks reported so far as metal-free catalysts in the same process.

## Experimental

### Materials and methods

**Synthesis of CTF1**–**5.** CTF materials have been synthesized via ionothermal synthesis in quartz glass ampules according to literature procedures [22]. In a general procedure, **CTF1**–**3** were prepared as follows: 3 g of the selected monomer (**I**, **II** or **III**) were thoroughly mixed and finely ground with 5 equiv of ZnCl_2_ within a glovebox and transferred into a quartz ampule (12 cm height and 3 cm diameter). After drying the material under vacuum for at least 3 h, the ampule was flame-sealed, placed inside a furnace and heated up to 400 °C with a heat rate of 10 °C·min^−1^. Afterwards, it was kept at 400 °C for 10 h before raising the temperature to 600 °C (second heating phase) and keeping the sample at that temperature for further 10 h. After cooling to room temperature, the ampules were opened (**caution:** after high-temperature treatment the ampules are under pressure, which is released during opening) and the monolithic solids were ground and thoroughly washed with water and diluted HCl (0.1 M). Finally, the solids were finely ground using a laboratory ball mill (Fritsch Pulverisette 23, 5 min, 30 Hz) to get black powders, which were carefully washed in sequence with water, diluted HCl, diluted NaOH, water and THF. At the end of each work-up, materials were dried under vacuum to constant weight (at least 12 h at 60 °C). Mixed CTFs (**CTF4**,**5**) were obtained following an identical procedure except for the use of a 50:50 molar ratio of the two starting monomers (DCI (**III**)/*p*-DCB (**I**) or DCI (**III**)/DCBP(**II**)) while keeping constant the 1:5 molar ratio between monomer(s) and ZnCl_2_ [21,71]. All materials (**CTF1**–**5**) were isolated in nearly quantitative yield (≥90% after work-up). To prevent bursting within the furnace, ampules were charged to a maximum of half of their volume.

**Elemental analyses** were performed using a Thermo FlashEA 1112 Series CHNS-O elemental analyzer and elemental average values for each sample were calculated over three independent runs.

**X-ray photoelectron spectroscopy (XPS)** measurements were performed in an ultrahigh vacuum (UHV) Thermo-VG scientific spectrometer equipped with a CLAM4 (MCD) hemispherical electron analyser. The Al Kα line (1486.6 eV) of a dual anode X-ray source was used as incident radiation. Survey and high-resolution spectra were recorded in constant pass energy mode (100 and 20 eV, respectively). Elemental semi-quantitative atomic percentages were calculated by fitting the spectra with mixed Gaussian–Lorentzian peaks applying tabulated sensitivity factors.

**Temperature-programmed oxidation (TPO-MS)** analyses were carried out on a Hiden Analytical CATLAB instrument coupled with a quadrupole mass spectrometer (detection limit = 2 × 10^−14^ Torr). In a typical analysis, 5–8 mg of CTF were charged in the sample holder and flushed at room temperature for 30 min under a stream of 10% O_2_ in Ar (flow rate: 25 mL/min). Afterwards, the temperature was raised up to 900 °C at a heating rate of 10 °C/min and the evolved volatile species (*m*/*z* 2 (H_2_), 18 (H_2_O), 28 (CO) and 44 (CO_2_)) were monitored through a mass spectrometer connected at the furnace outlet.

**X-ray powder diffraction (PXRD)** qualitative measurements were carried out with a Panalytical X’PERT PRO powder diffractometer equipped with a mirror on the incident beam, a beam knife and a PIXcel^©^ solid state detector in the 4–60° 2θ region, operating with Cu Kα radiation (λ = 1.5418 Å). Anti-scatter slits were used both on the incident (0.25° and 0.5° divergence) and the diffracted (7.5 mm height) beam.

**Gas adsorption measurements.** In a similar manner as described before [22], nitrogen physisorption experiments were conducted on a Micromeritics ASAP 2010 instrument. Samples were degassed for at least 15 h at 150 °C using a FloVacDegasser. Static volumetric measurements were carried out at 77 K. The empty volume of the cell was determined with helium. The specific surface area (SSA) for each sample was determined by the Brunauer–Emmet–Teller method (BET) using data points at a relative pressure *p*/*p*_0_ between 0.05 and 0.3. The total pore volume was determined at a relative pressure of 0.98. The pore size distribution was calculated via Micro-Active (version 1.01) using the density functional theory (DFT) N_2_ model for slit geometry at optimal goodness of fit vs regularization (0.01) values for both RMS error of fit and roughness of distribution. The cumulative pore volume at the pore width of 2 nm was used to determine the micropore volume of the samples.

**Low pressure adsorption isotherms** were recorded on an ASAP 2020 Micromeritics instrument after activation of CTF samples at 200 °C for 12 h. CO_2_ adsorption isotherms were recorded at 273 K and 298 K up to 1.2 bar, while N_2_ adsorption isotherms for the determination of the CO_2_/N_2_ selectivity were measured at 298 K up to 1.2 bar.

The isosteric heat of adsorption (*Q*_st_) was calculated from the measured CO_2_ isotherms at 273 and 298 K using a variant of the Clausius–Clapeyron equation (Equation 1) [51,63]:

[1] $$\ln\left(\frac{P_{1}}{P_{2}}\right)=Q_{\text{st}}\times \frac{T_{2}-T_{1}}{R\times T_{1}\times T_{2}}$$

where *P_n_* (*n* = 1 or 2) is the pressure value for isotherm *n*; *T_n_* (*n* = 1 or 2) is the temperature value for isotherm *n*; *R* is the gas constant, *R* = 8.314 J·K^−1^·mol^−1^. CO_2_/N_2_ selectivity was calculated on the basis of the Henry model, taking into account the initial slopes of the adsorption isotherms (Supporting Information File 1, Figure S7). The IAST selectivity for a 15:85 CO_2_/N_2_ mixture at a total pressure of 1 bar was determined from Equation 2:

[2] $$S_{{\text{CO}}_{\text{2}}/{\text{N}}_{\text{2}}}=\frac{{\left[\chi_{{\text{CO}}_{\text{2}}}/\chi_{{\text{N}}_{\text{2}}}\right]}_{\text{ads}}}{{\left[\chi_{{\text{CO}}_{\text{2}}}/\chi_{{\text{N}}_{\text{2}}}\right]}_{\text{mix}}}$$

where (χ*_i_*)_ads_ represent the adsorbed molar fractions of the two gases [72] as derived from the application of the free python software pyIAST (https://github.com/CorySimon/pyIAST) to the experimental N_2_ and CO_2_ isotherms of **CTF1**–**5** collected at 298 K, while (χ*_i_*)_mix_ are the molar fractions of the two gases in the starting mixture (0.85 and 0.15 for N_2_ and CO_2_, respectively). A BET (CO_2_) and a Henry (N_2_) model were employed for the isotherms fitting. For a detailed explanation of these methods, see the pyIAST webpage and documentation.

### Catalytic oxygen- and steam-free direct dehydrogenation of ethylbenzene to styrene

The catalytic reaction was carried out in a fixed-bed continuous flow reactor under atmospheric pressure. 300 mg of CTF were loaded into a quartz fritted disk located inside a tubular quartz reactor (i.d. × length 8 × 800 mm). Helium was fed into the reactor (30 mL·min^−1^) through a mass flow controller (BROOKS MFC) and passed through a glass evaporator filled with liquid EB maintained at constant temperature with a regulated thermal bath. The reaction system was heated to 550 °C and kept for 2 h under He. The reactant flow (2.8 vol % EB diluted in He, total flow rate of 30 mL·min^−1^) was then fed to the reactor. The reactant and the products (styrene (ST), benzene (BZ) and toluene (TOL)) getting out from the reactor were analyzed on line with a PERICHROM (PR 2100) gas chromatograph equipped with a flame detector (FID) and a previously calibrated CP WAX S2CB column. In order to avoid any possible condensation of the reactant or the products, all the tube lines were wrapped with a heating wire kept at 110 °C. The ethylbenzene conversion (*X*_EB_) and styrene selectivity (*S*_ST_) were evaluated using Equation 3 and Equation 4:

[3] $$X_{\text{EB}}=\frac{F_{0}C_{\text{EB}\text{,inlet}}-FC_{\text{EB}\text{,outlet}}}{F_{0}C_{\text{EB}\text{,inlet}}}\times 100\%$$

[4] $$S_{\text{ST}}=\frac{C_{\text{ST}\text{,outlet}}}{C_{\text{ST}\text{,outlet}}+C_{\text{TOL}\text{,outlet}}+C_{\text{BZ}\text{,outlet}}}\times 100\%$$

where *F* and *F*_0_ are the flow rates of the outlet and inlet, respectively, while *C*_EB_, *C*_ST_, *C*_TOL_ and *C*_BZ_ correspond to the concentration of ethylbenzene, styrene, toluene and benzene. The carbon balances amounted to about 100% in all trials.

## Supporting Information

Complementing material characterization, such as CHN elemental analysis, nitrogen adsorption−desorption isotherms, differential pore volume distributions, survey spectra and N 1s, O 1s core region XPS analyses, low-pressure CO_2_ adsorption–desorption isotherms, heats of adsorption (*Q*_st_), CO_2_ and N_2_ adsorption isotherms at 298 K, TPO and PXRD analyses.

File 1 Additional experimental data.
