# Supplementary material for: Playing with covalent triazine framework tiles for improved CO2 adsorption properties and catalytic performance
Source: Beilstein J Nanotechnol. 2019 Jun 12;10:1217–27. doi: 10.3762/bjnano.10.121 (PMC6604744; doi:10.3762/bjnano.10.121)
Supplement: File 1 — Additional experimental data. [file Beilstein_J_Nanotechnol-10-1217-s001.pdf]

BEILSTEIN JOURNAL OF NANOTECHNOLOGY

Supporting Information

for

Playing with covalent triazine framework tiles for improved CO_2 adsorption properties and catalytic performance

Giulia Tuci, Andree Iemhoff, Housseinou Ba, Lapo Luconi, Andrea Rossin, Vasiliki Papaefthimiou, Regina Palkovits, Jens Artz, Cuong Pham-Huu and Giuliano Giambastiani

Beilstein J. Nanotechnol. **2019,** *10,* 1217–1227. doi:10.3762/bjnano.10.121

Additional experimental data

License and Terms: This is a supporting information file under the terms of the Creative Commons Attribution License (http://creativecommons.org/licenses/by/4.0). Please note that the reuse, redistribution and reproduction in particular requires that the authors and source are credited.
The license is subject to the *Beilstein Journal of Nanotechnology* terms and conditions: (https://www.beilstein-journals.org/bjnano)

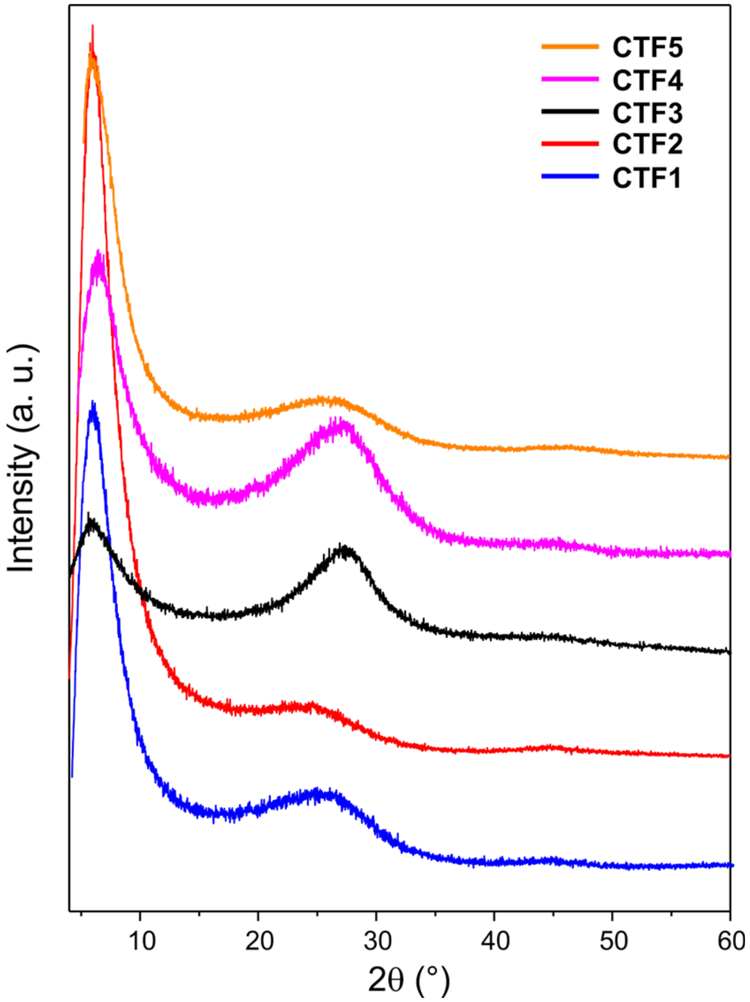

Figure S1: Powder X-ray diffraction analyses of **CTF1–5**, showing their amorphous nature.

Table S1: CHN Elemental Analyses of **CTF1–5** samples. All values are given as an average value over three independent runs.

Sample	N wt %	C wt %	H wt %
**CTF1**	7.5	65.2	2.2
**CTF2**	3.6	77.9	1.2
**CTF3**	29.1	40.0	2.6
**CTF4**	18.1	50.3	2.4
**CTF5**	11.4	61.8	2.0

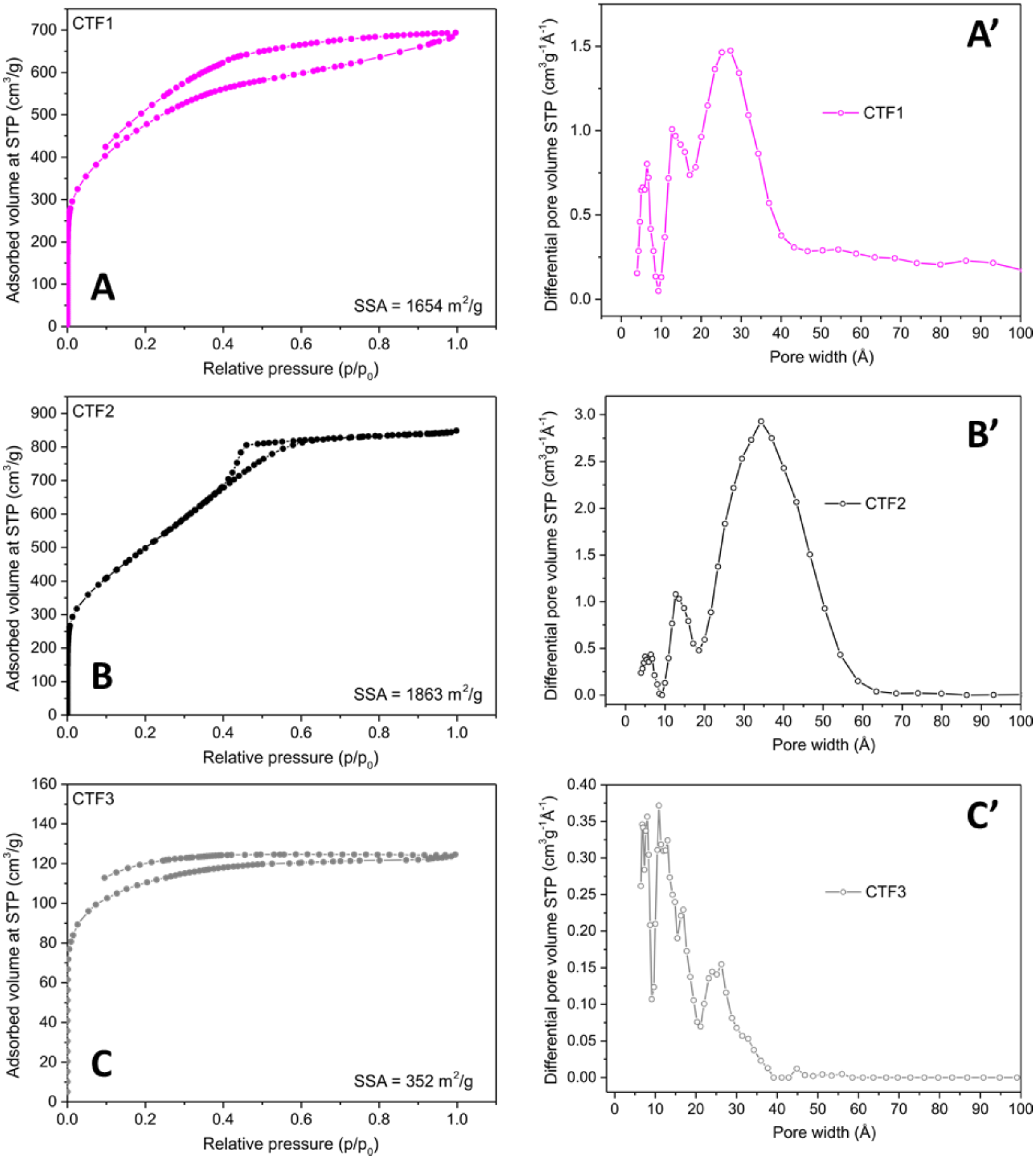
CTF1
A
SSA = 1654 m2/g
Adsorbed volume at STP (cm3/g)
Relative pressure (p/p0)
A’
CTF1
Differential pore volume STP (cm3g-1Å-1)
Pore width (Å)
CTF2
B
SSA = 1863 m2/g
Adsorbed volume at STP (cm3/g)
Relative pressure (p/p0)
B’
CTF2
Differential pore volume STP (cm3g-1Å-1)
Pore width (Å)
CTF3
C
SSA = 352 m2/g
Adsorbed volume at STP (cm3/g)
Relative pressure (p/p0)
C’
CTF3
Differential pore volume STP (cm3g-1Å-1)
Pore width (Å)

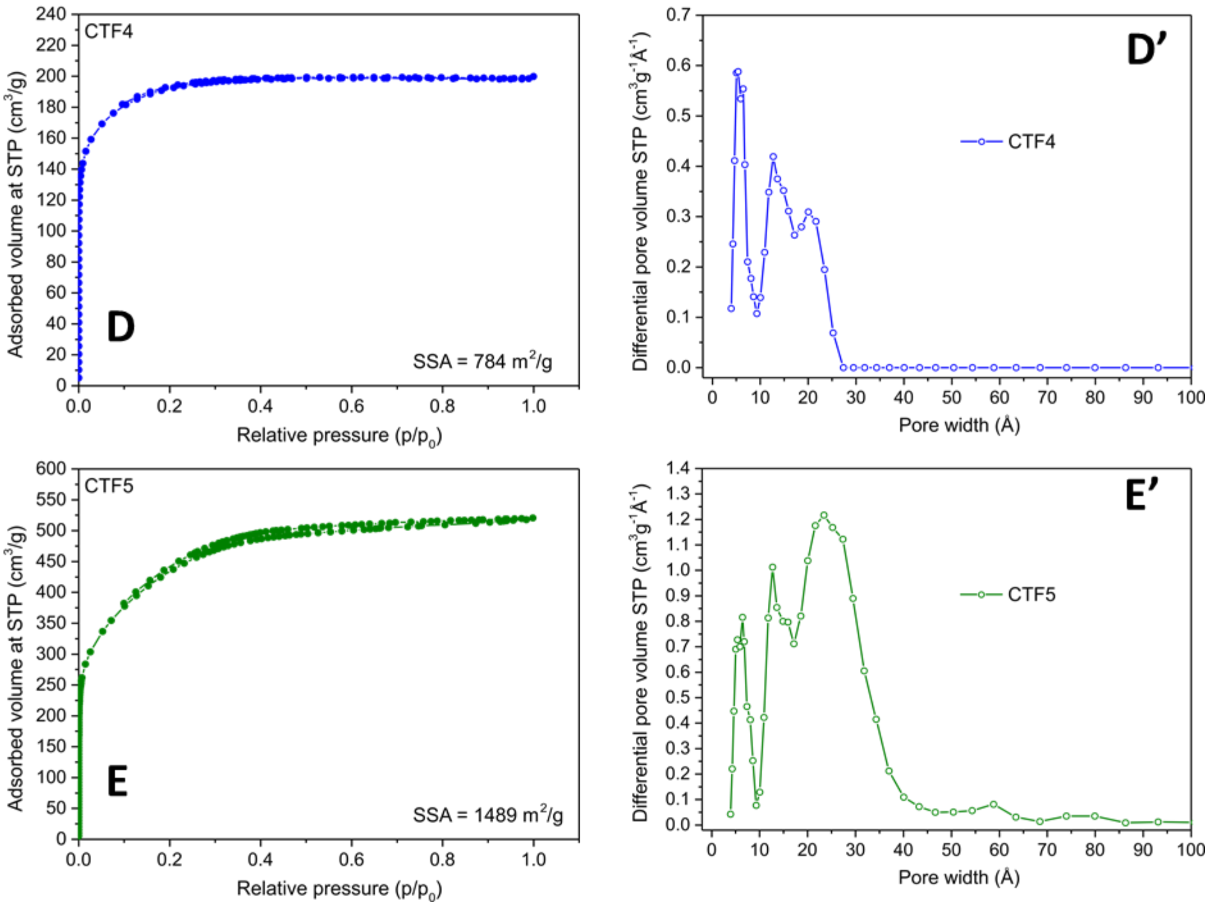

Figure S2: Nitrogen adsorption−desorption isotherms of **CTF1–5** (**A–E**) recorded at 77 K and related pore size distribution evaluated through a NLDFT pore modelling (**A’–E’**). Adsorption-desorption branches of the isotherm recorded for **CTF3** (C) do not close due to incomplete desorption. This effect is likely attributed to a strong material adsorption of N_2 within its very narrow slit-like pores.

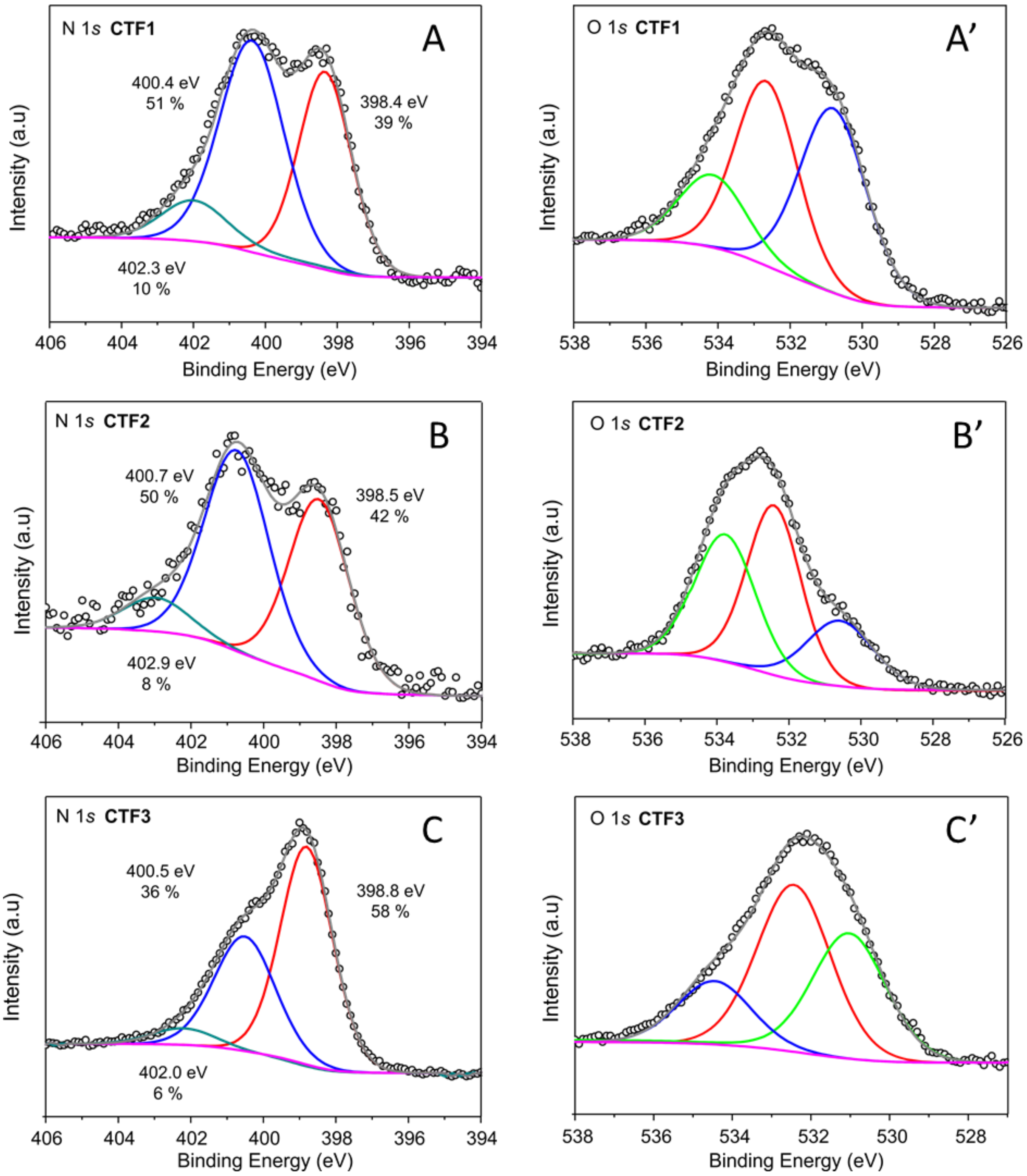
N 1s CTF1
A
400.4 eV
51 %
398.4 eV
39 %
402.3 eV
10 %
O 1s CTF1
A'
N 1s CTF2
B
400.7 eV
50 %
398.5 eV
42 %
402.9 eV
8 %
O 1s CTF2
B'
N 1s CTF3
C
400.5 eV
36 %
398.8 eV
58 %
402.0 eV
6 %
O 1s CTF3
C'
Intensity (a.u)
Binding Energy (eV)

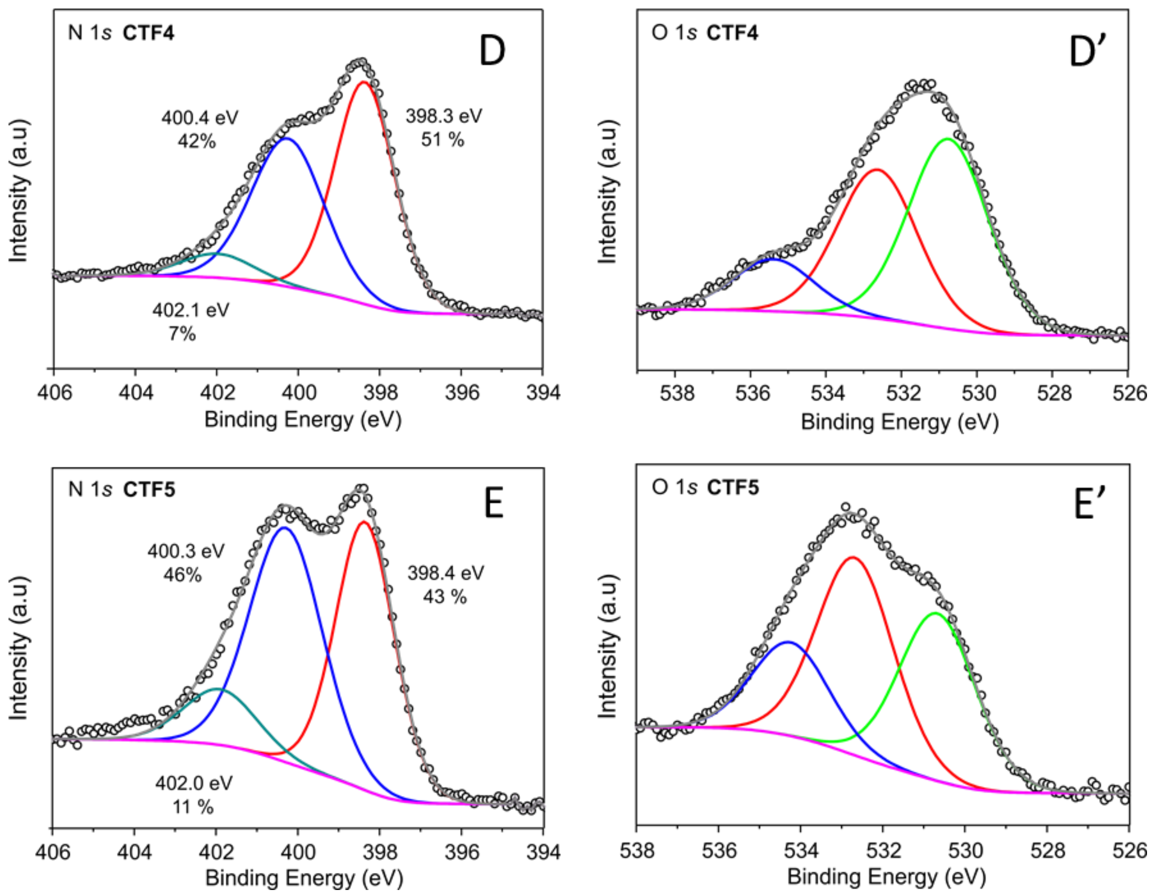

Figure S3: XPS N 1s (**A–E**) and O 1s (**A’–E’**) core level regions of **CTF1–5** along with their relative curves fittings

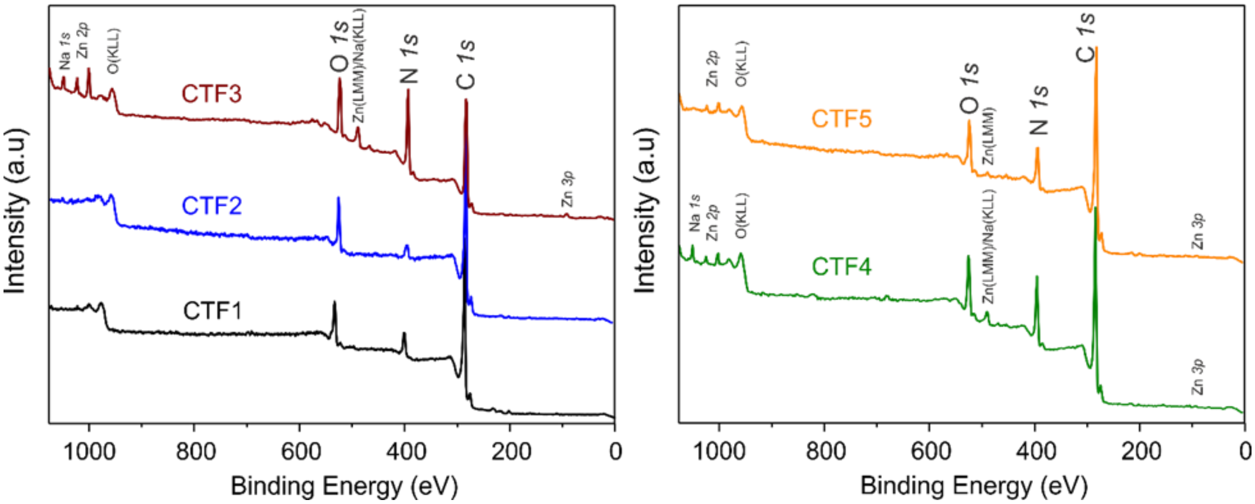

Figure S4: XPS survey spectra recorded for all CTF samples. Zn residues (traces) from the ionothermal synthesis not completely removed from the carefully purified samples during the material work-up are present in **CTF3–5**. The higher the micropore percentage of the CTF sample the higher is the level of Zn contamination (**CTF3** > **CTF4** > **CTF5**). It is important to note that the role of Zn residues with respect to the material performance in EB DDH has already been ruled out in one of our recent publication in the field [1]. Moreover, in mixed oxide-based catalysts derived from hydrotalcites, the higher the Zn content in the catalyst the lower the catalytic performance in DDH process [2]. Overall, Zn residues simply play as innocent spectators with respect to the DDH process.

The % content of all elements was calculated using the corresponding core level peaks properly normalized to the photoemission cross section and assuming a homogeneous distribution arrangement model, and results are given in the table below.

Table of Figure S4: Surface atomic ratios of all elements measured by XPS.

SAMPLE	%C	%O	%Zn	%N	%Na	C/N
**CTF1**	83.7	8	0	8.3	0	10.1
**CTF2**	87.9	8.8	0	3.3	0	26.2
**CTF3**	64.3	10	0.8	24.1	0.8	2.7
**CTF4**	71.4	10.9	0.3	16.7	0.6	4.3
**CTF5**	79.1	9.6	0.2	11.1	0.1	7.1

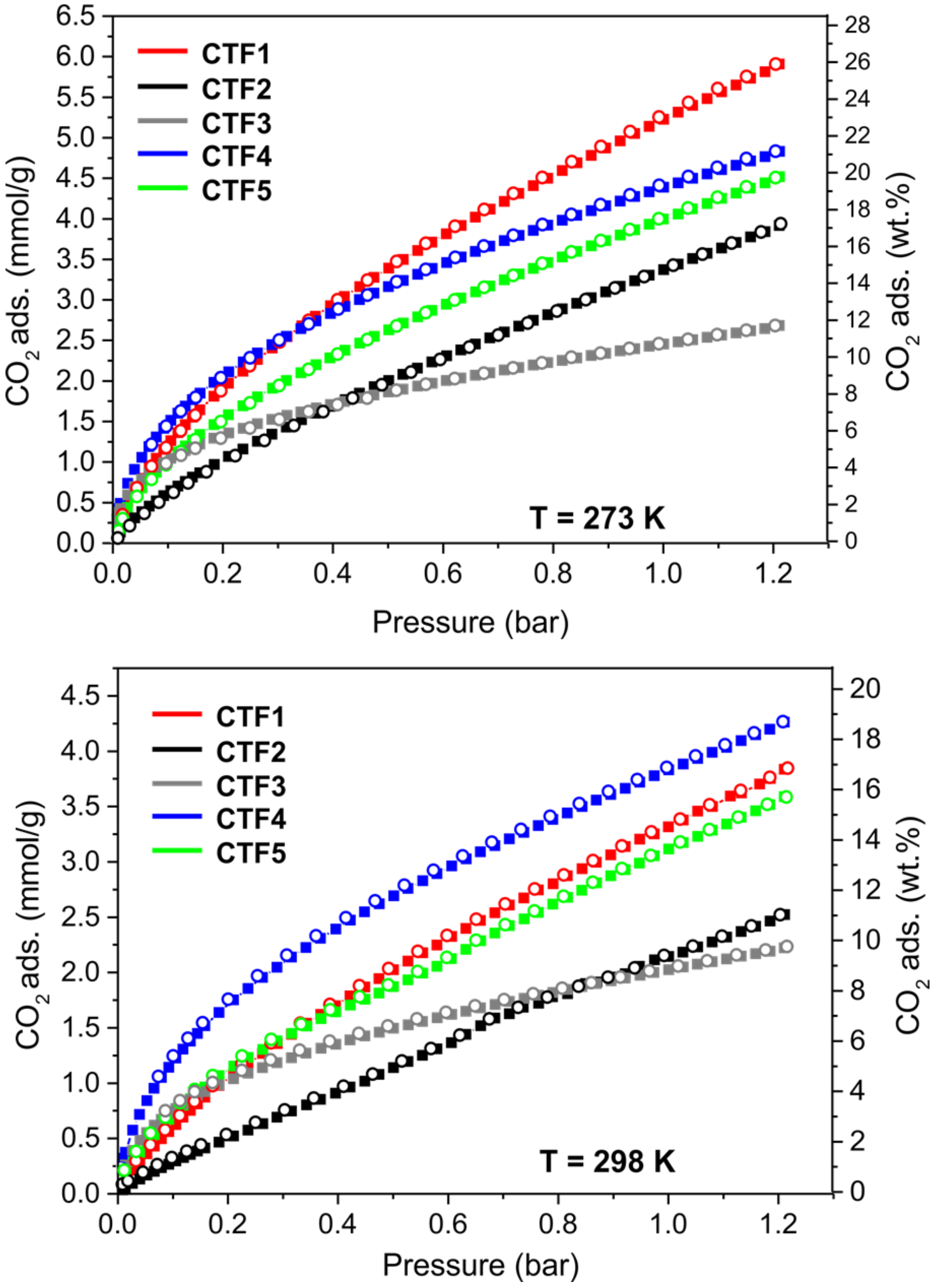

Figure S5: Low-pressure CO_2 adsorption-desorption isotherms for **CTF1–5** at **A**) *T* = 273 K and **B**) *T* = 298 K. (solid squares = adsorption isotherms; empty round symbols = desorption isotherms).

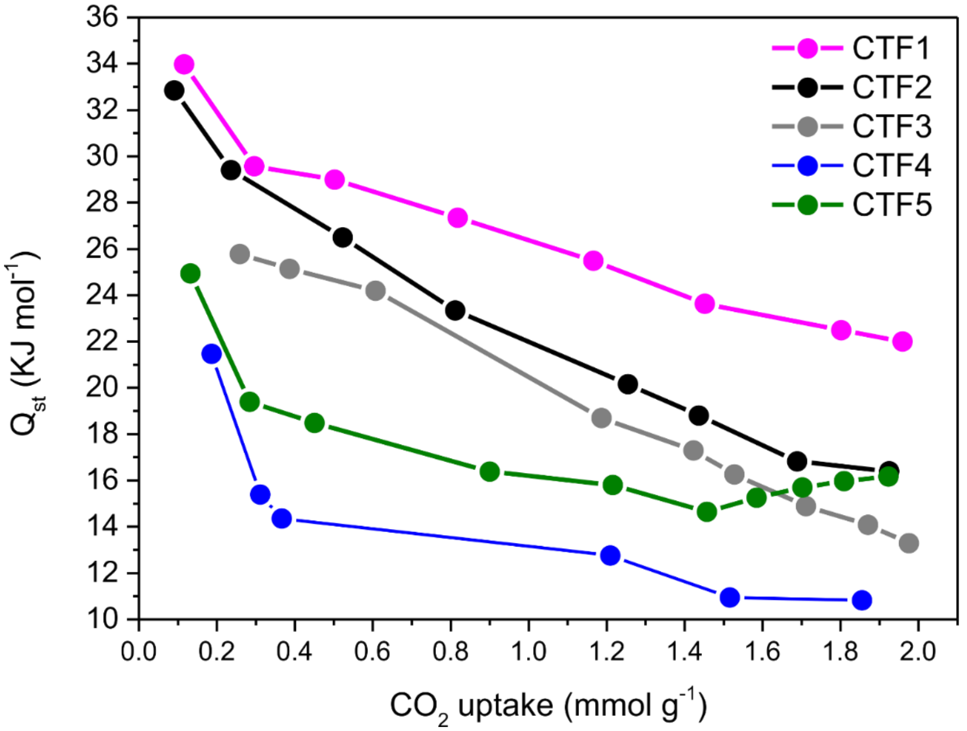

Figure S6: Heats of adsorption (Q_{st}) for **CTF1–5** measured from the CO_2 isotherms recorded at 273 and 298 K.

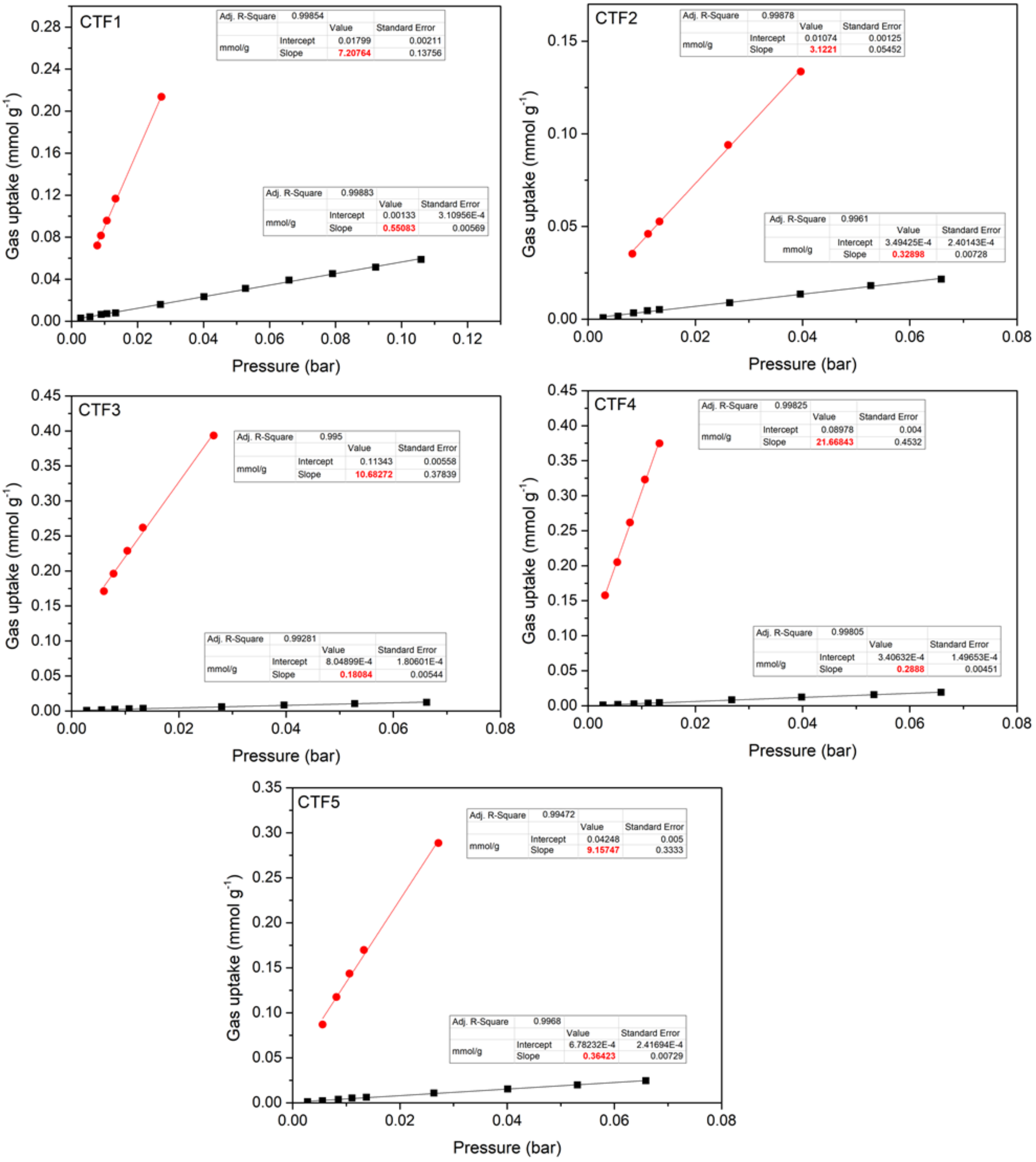

Figure S7: Comparison of the N_2 (black) and CO_2 (red) isotherms at 298 K in the 0–0.1 bar interval for the estimation of the CO_2 vs N_2 selectivity of **CTF1–5** samples through the Henry method.

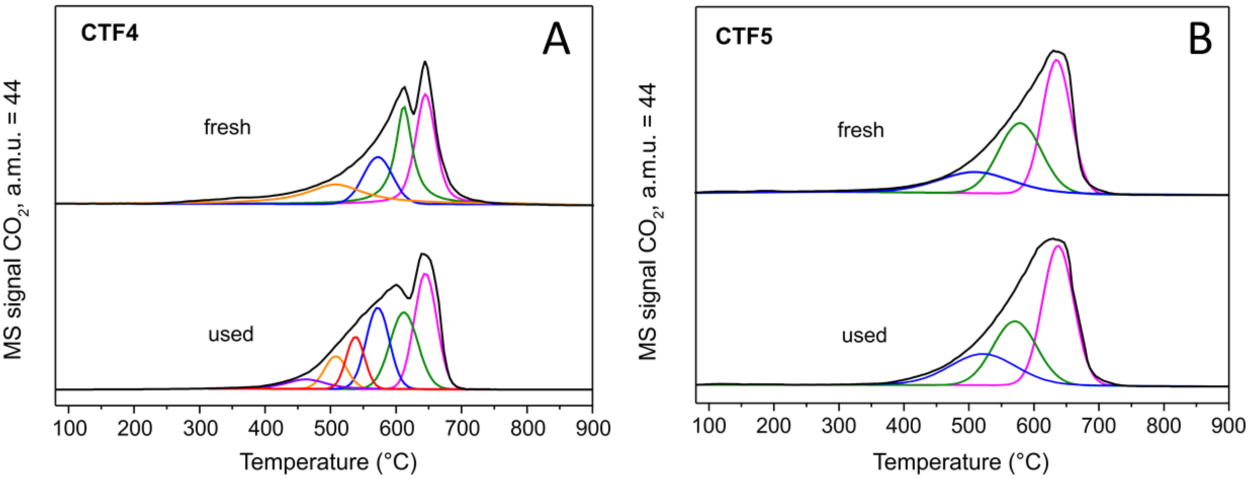

Figure S8: CO_2 evolution as recorded from the temperature programmed oxidation (TPO) analyses on (**A**) **CTF4** and (**B**) **CTF5** before (top curves) and after (bottom curves) their use in DDH.

References

1. Tuci, G.; Pilaski, M.; Ba, H.; Rossin, A.; Luconi, L.; Caporali, S.; Pham-Huu, C.; Palkovits, R.; Giambastiani, G., *Adv. Funct. Mater.* **2017**, *27*, 1605672. doi:10.1002/adfm.201605672
2. Balasamy, R. J.; Tope, B. B.; Khurshid, A.; Al-Ali, A. A. S.; Atanda, L. A.; Sagata, K.; Asamoto, M.; Yahiro, H.; Nomura, K.; Sano, T.; Takehira, K.; Al-Khattaf, S. S., *Appl. Catal., A* **2011**, *398*, 113-122. doi:10.1016/j.apcata.2011.03.023
